# Phylloseptin-1 is Leishmanicidal for Amastigotes of *Leishmania amazonensis* Inside Infected Macrophages

**DOI:** 10.3390/ijerph17134856

**Published:** 2020-07-06

**Authors:** Selma A. S. Kückelhaus, Daniela Sant’Ana de Aquino, Tatiana K. Borges, Daniel C. Moreira, Luciana de Magalhães Leite, Maria Imaculada Muniz-Junqueira, Carlos S. Kückelhaus, Gustavo A. Sierra Romero, Maura V. Prates, Carlos Bloch, José Roberto S. A. Leite

**Affiliations:** 1 Núcleo de Pesquisa em Morfologia e Imunologia Aplicada, NuPMIA, Área de Morfologia, Faculdade de Medicina, FM, Universidade de Brasília, Brasília, DF 70910900, Brazil; danisantana.aquino@gmail.com (D.S.d.A.); moreiradc@unb.br (D.C.M.); medvet.luciana@gmail.com (L.d.M.L.); jrsaleite@gmail.com (J.R.S.A.L.); 2 Laboratório de Imunologia Celular, Núcleo de Pesquisa em Morfologia e Imunologia Aplicada, NuPMIA, Faculdade de Medicina, FM, Universidade de Brasília, Brasília, DF 70910900, Brazil; tatianakarlab@gmail.com (T.K.B.); mimjunqueira@gmail.com (M.I.M.-J.); 3 Laboratório de Leishmaniose, Núcleo de Medicina Tropical, Universidade de Brasília, Brasília, DF 70910900, Brazil; cscarlossantos@gmail.com (C.S.K.); gromero@unb.br (G.A.S.R.); 4 Laboratório de Espectrometria de Massa, LEM, EMBRAPA Recursos Genéticos e Biotecnologia, Brasília, DF 70770900, Brazil; maura.prates@embrapa.br (M.V.P.); carlos.bloch@embrapa.br (C.B.J); 5 Bioprospectum, Lda, Parque Tecnológico da Universidade do Porto, UPTEC, 4200135 Porto, Portugal

**Keywords:** antimicrobial peptides, cytokine, leishmaniasis, oxidative stress

## Abstract

Leishmania protozoans are the causal agents of neglected diseases that represent an important public health issue worldwide. The growing occurrence of drug-resistant strains of *Leishmania* and severe side effects of available treatments represent an important challenge for the leishmaniases treatment. We have previously reported the leishmanicidal activity of phylloseptin-1 (PSN-1), a peptide found in the skin secretion of *Phyllomedusa* *azurea* (=*Pithecopus azureus*), against *Leishmania* *amazonensis* promastigotes. However, its impact on the amastigote form of *L. amazonensis* and its impact on infected macrophages are unknown. In this work, we evaluated the effects of PSN-1 on amastigotes of *L. amazonensis* inside macrophages infected in vitro. We assessed the production of hydrogen peroxide and nitric oxide, as well as the levels of inflammatory and immunomodulatory markers (TGF-β, TNF-α and IL-12), in infected and non-infected macrophages treated with PSN-1. Treatment with PSN-1 decreased the number of infected cells and the number of ingested amastigotes per cell when compared with the untreated cells. At 32 µM (64 µg/mL), PSN-1 reduced hydrogen peroxide levels in both infected and uninfected macrophages, whereas it had little effect on NO production or TGF-β release. The effect of PSN-1 on IL-12 and TNF-α secretion depended on its concentration, but, in general, their levels tended to increase as PSN-1 concentration increased. Further in vitro and in vivo studies are needed to clarify the mechanisms of action of PSN-1 and its interaction with the immune system aiming to develop pharmacological applications.

## 1. Introduction

Protozoans of the *Leishmania* genus are the causal agents of important but neglected tropical diseases that affect 0.7–1 million people each year worldwide [1,2,3,4]. Leishmaniases are classified as cutaneous, mucosal and visceral, depending on the clinical manifestations. The first form is characterized by self-healing cutaneous ulcers, the second by severe disfiguration of mucosal regions and the last by its effect on internal organs such as liver and spleen [5]. For a long time, the administration of pentavalent antimony (SbV) has been the leading treatment for leishmaniasis [6], regardless of its severe side effects [7]. Indeed, the treatment of leishmaniases is associated with undesirable side effects, including toxicity, high cost, long periods of treatment and inconvenient route of administration [8]. Noteworthy, parasite resistance is an important issue and, as it happens for bacteria, the occurrence of drug-resistant strains of protozoa represents an important challenge for the leishmaniases treatment and public health. These factors motivate the development of novel anti-leishmanial treatments.

**Figure 1:**
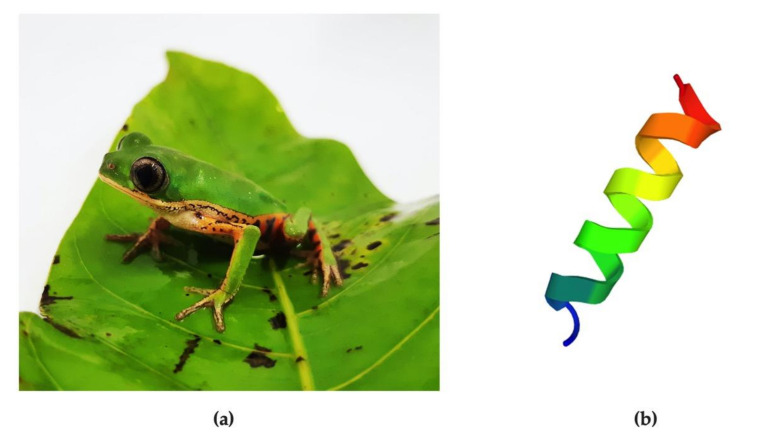
*Phyllomedusa azurea* (renamed for *Pithecopus azureus* (Cope, 1862)) (Anura: Hylidae) and the phylloseptin-1 (PSN-1) peptide. (**a**) Adult specimen of *P. azurea*, whose skin secretion was the first source where PSN-1 was identified (Photo: Eder A. Barbosa). (**b**) Tridimensional representation of PSN-1 predicted by PEP-FOLD 3.5/De novo peptide structure prediction [23].

Antimicrobial peptides have emerged as attractive alternatives for the development of new drugs. Peptides present relative low toxicity [9,10] and a broad antibiotic action against pathogens, including viruses [11], bacteria [12], fungi [13] and protozoa [14,15]. The amphibians’ skin is a natural source of such peptides; therefore, many research groups have investigated it, looking for novel bioactive molecules to treat human diseases. In the case of leishmaniasis, we have previously reported that phylloseptin-1 (PSN-1), formerly known as PS-1 [16], has a strong activity against *Leishmania amazonensis* promastigotes [10]. This cationic peptide (at physiological pH) was first isolated from the cutaneous secretion from *Phyllomedusa azurea* (renamed for *Pithecopus azureus* (Cope, 1862)) (Anura: Hylidae) and belongs to the phylloseptins family (Figure 1a). PSN-1 has 19 amino acid residues (FLSLIPHAINAVSAIAKHN-NH_2_^)^ and a molecular weight of 2016 Da. While PSN-1 shows low toxicity and negligible hemolytic effects [17], there are several reports about its antimicrobial activities. For instance, it displays significant activity against *Plasmodium falciparum* [17], gram-positive bacteria [9], gram-negative bacteria [9], bacterial biofilm [18] and yeast [19].

Notably, PSN-1 is able to strongly suppress the growth of three leishmania species, *Leishmania infantum*, *L. major* and *L. braziliensis* in their promastigote stage [17,20]. Since *Leishmania* amastigotes are the main form found inside macrophages during human infections, it is urgent to evaluate the effect of PSN-1 on this form of *L. amazonensis* to better understand the potential role of PSN-1 as an anti-leishmanial drug. In this study, we assessed the effects of the PSN-1 peptide on amastigote-infected peritoneal macrophages. We evaluated infection index, hydrogen peroxide production, nitric oxide generation and levels of the immunomodulatory IL-12 and TGF-β, and the level of the inflammatory TNF-α. This approach is likely to be more clinically relevant than that using promastigotes, since this is the form present inside macrophages, which are the main cells involved in the pathogenesis of the leishmaniases.

## 2. Materials and Methods

### 2.1. Phylloseptin-1 Synthesis and Isolation

Amidated PSN-1 (FLSLIPHAINAVSAIAKHN-NH_2_; Figure 1) was synthesized on a Prelude Peptide Synthesizer (Protein Technologies Inc. Tucson, AZ, USA). F-moc amino acids and F-moc-Rink-Amide-MBHA resin were purchased from Peptides International (Louisville, KY, USA). After synthesis, the peptide was cleaved from the resin by a cocktail containing trifluoracetic acid (TFA), triisopropylsilane (TIS) and water, 95:5:5 (v/v/v). The product was precipitated by adding cooled diisopropyl ether, and the resulting crude extract was freeze-dried. The thin-white powder obtained was mass analyzed (MicrOTOF-Q II, Bruker Daltonics, Bremen, Germany) in order to check the peptide integrity.

Aliquots of 10 mg from the lyophilized and filtered product were injected into a preparative Jupiter C_18_ column (Phenomenex, Torrance, CA, USA) coupled to an HPLC Prominence System (Shimadzu Corporation, Kyoto, Japan) to isolate the active peptide fraction from by-products. The peptide elution was monitored at 216 and 280 nm, under a step-optimized gradient of acetonitrile and water (TFA 0.1%); from 0% to 100% acetonitrile. Molecular mass and PSN-1 purity were checked by MALDI/TOF-TOF-MS on an Ultraflex II (Bruker Daltonics, Bremen, Germany).

Stock solutions of PSN-1 (prepared in phosphate-buffered saline) had their concentration checked and adjusted using ultraviolet spectrophotometry [21,22]. Cells were treated with PSN-1 at the final concentrations of 2, 16 or 64 µg/mL, which correspond to 1, 8 or 32 µM, respectively. These concentrations were chosen based on our previous work [17].

### 2.2. Leishmania amazonensis

The isolate MHOM/BR/pH8 of *Leishmania amazonensis* was obtained from the Laboratory of Leishmaniasis of the Institute of Tropical Medicine, University of Brasilia, Brazil. It was cryopreserved in liquid nitrogen until transfer to an NNN medium and cultured at 24 °C for 48 h. In sequence, a small aliquot was added to Schneider culture medium, supplemented with 20% heat-inactivated fetal calf serum (Sigma Aldrich, St. Louis, MO, USA) and gentamicin sulphate (40 mg/mL; Schering Plough, São Paulo, Brazil), and cultured until the log phase was reached. The axenic amastigotes were obtained by maintaining the suspension incubated at 37 °C for 96 h [24]. After monitoring different cell forms by microscopic analyses, only amastigote *L. amazonensis* cells were used to infect macrophages.

### 2.3. Animals

Female adult Swiss mice (*n* = 8) (Faculty of Medicine, University of Brasilia, Brazil) at 2 months of age and weighting 35 ± 5 g were maintained at 12 h dark/light cycles. Animals had access to balanced food and water ad libitum and were kept at room temperature until experiments. The Animal Research Ethical Committee of the Brasília University approved the experimental protocol (process number 51002/2013).

### 2.4. Macrophage Infection

Mouse macrophages were obtained by washing the peritoneal cavity with 10 mL of cold phosphate-buffered saline (PBS, pH 7.2). Cells were washed with cold PBS (200× *g*, 10 min), quantified in hemocytometer and suspended into cold RPMI 1640 medium (Sigma, St. Louis, MO, USA), pH 7.2, supplemented with 20 mM HEPES (Sigma), 2 mM glutamine (Sigma) and 2.5 mg/dl gentamycin. Viability was assessed in hematocytometer with 0.05% nigrosin solution in PBS, pH 7.2; viability was always higher than 97%. Then, samples of 2 × 10^5^ macrophages in RPMI 1640 were placed, in duplicate, on 13 mm-diameter glass coverslips in 24-well plastic plates (Linbro, Corning, NY, USA), incubated for 2 h in a wet chamber, at 37 °C, in the presence of 5% CO_2_ in air to allow adherence of macrophages onto the glass. Then, the coverslips were rinsed with PBS, pH 7.2, at 37°C, to eliminate non-adhered cells. Adherent cells (>98% macrophages) were incubated with 10^6^ axenic amastigotes forms of *L. amazonensis* per well, suspended in 500 µL of RPMI 1640, supplemented with 10% inactivated fetal calf serum (Gibco), in a wet chamber for 8 h at 37 °C, in 5% CO_2_ in air. The preparations were then rinsed with PBS at 37 °C to eliminate non-phagocytosed *Leishmania*. After that, infected macrophages were incubated with different concentrations of PSN-1 (0, 1, 8 or 32 µM) for 2 h, rinsed with PBS, fixed with absolute methanol and stained with 10% buffered Giemsa solution (pH 7.2). The number of amastigotes ingested by macrophages and the percentage of infected cells were assessed by optical microscopy. Parasites were considered as dead when their typical morphology was altered, and their integrity was lost. The infection index was calculated as the average number of ingested amastigotes multiplied by the percentage of macrophages engaged in phagocytosis [25]. Images of cells were obtained using a Zeiss Axio Lab.A1 microscope connected to an Axiocam ERc 5s image capture system (Carl Zeiss, Oberkochen, Germany).

### 2.5. Nitric Oxide Production as Estimated by Nitrite Measurement

Peritoneal cells were collected as described in Section 2.4, seeded at 2 × 10^5^ cells per well in 96-well plates in 200 µL of RPMI 1640 incomplete medium and incubated in a wet chamber at 37 °C and 5% CO_2_ for 2 h. Then, wells were washed three times with PBS at 37 °C, and 10^6^ amastigotes of *Leishmania* were added per well.

Plates were incubated under the previous conditions for 8 h. Wells were washed three times with PBS at 37 °C and then treated with 0, 1, 8 or 32 µM PSN-1 for 24 h. Lipopolysaccharides from *E. coli* (055:b5, Sigma-Aldrich) at 20 ng/mL in PBS were used as a positive control. Plates were centrifuged at 200× *g* for 10 min and 100 µL of the supernatants were transferred to another plate and incubated *v/v* with Griess reagent (1% sulphanilamide/0.1% N-1-naphthylethylene diaminedihydrochloride/2.5% H_3_PO_4_) at room temperature for 10 min. [26]. Then, the plate was read at 540 nm, (SpectraMax^®^ Plus 384; Molecular Devices, Sunnyvale, CA, USA). Blank was done in air. Results were expressed as µM NO_2_^-^.

### 2.6. Hydrogen Peroxide Production

Peritoneal cells were collected and incubated with *Leishmania* amastigotes as described in Section 2.5. Macrophages were then washed and incubated with 0, 1, 8 or 32 µM PSN-1 in 140 µL of a solution containing 5.5 mM dextrose, 0.5 mM phenol red and 19 U/mL of horseradish peroxidase type 2 RZ 1.3 (Sigma, St. Louis, MO, USA) and 100 nM phorbol myristate acetate (PMA) (Sigma, St. Louis, MO, USA), for 1 h. The reaction was stopped by adding 10 µL of 1 M NaOH/well. The plate was read at 620 nm in a microplate reader (SpectraMax^®^ Plus 384; Molecular Devices, Sunnyvale, CA, USA) [27]. Blank was done in air. Results were expressed as µM H_2_O_2_/2 × 10^5^ macrophages/h.

### 2.7. Cytokines

Levels of transforming growth factor beta (TGF-β), tumor necrosis factor alpha (TNF-α), and interleukin-12 (IL-12) were assessed in supernatant of the cultures of peritoneal cells infected with *Leishmania* amastigotes and treated with PSN-1 (0, 1, 8 or 32 µM) for 2 h, as described above, using commercially available ELISA kits (eBioscience, San Diego, CA, USA) as instructed by the manufacturer. The colorimetric reaction was read at a wavelength of 450 nm in a microplate reader, (SpectraMax^®^ Plus 384; Molecular Devices, Sunnyvale, CA, USA). Blank was done in air.

### 2.8. Statistical Analysis

Infection parameters results were analyzed using one-way ANOVA followed by Tukey’s multiple comparison test. For the other measurements, data were analyzed using one-way ANOVA followed by Tukey’s multiple comparisons test separately among infected or uninfected groups, or Kruskal–Wallis test followed by Dunn’s multiple comparisons test for data distributed non-parametrically. Infected and uninfected groups were compared pairwise for each PSN-1 concentration using two-tailed unpaired *t*-test, or Mann–Whitney test. All data were tested for normality using the Kolmogorov–Smirnov test and with Bartlett test for equal variances. For all analyses, *p* values lower than 0.05 were considered to indicate statistically significant differences. Statistical analyses and graphs were prepared using GraphPad Prism version 8 (GraphPad Software, La Jolla, CA, USA).

## 3. Results

**Figure 2:**
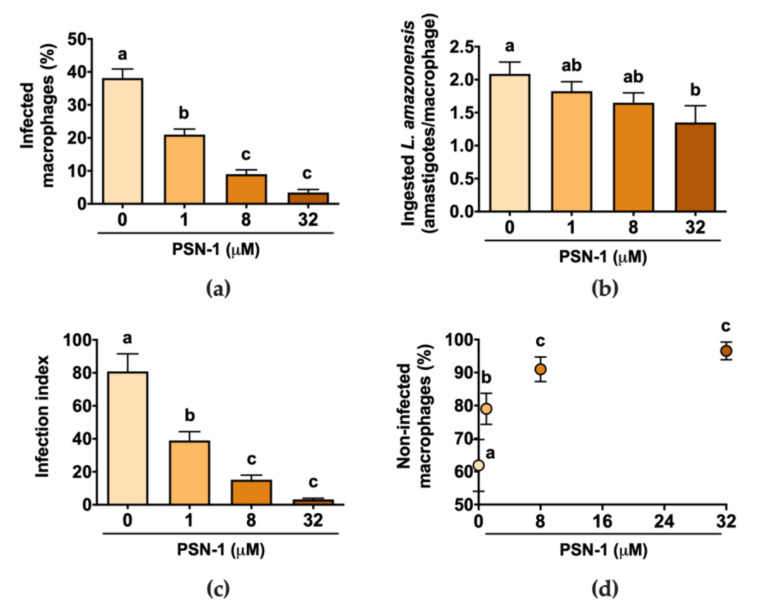
Effect of phylloseptin-1 (PSN-1) on axenic amastigotes of *Leishmania amazonensis* inside infected peritoneal macrophages. Cells were treated with different concentrations (0, 1, 8 or 32 μM) of PSN-1 for 2 h. (**a**) Percentage of infected macrophages, (**b**) number of amastigotes ingested by each macrophage, (**c**) index of infection and (**d**) number of non-infected macrophages (%) versus PSN-1 concentration. Data are presented as mean ± SEM, except for (**d**) where error bars stand for SD (*n* = 8 repeated experiments). Different letters (i.e., groups that do not share any letter) indicate significant differences (one-way ANOVA followed by Tukey’s multiple comparisons test; *p* < 0.05).

Incubation of amastigote-infected macrophages with different concentrations of PSN-1 decreased the percentage of infected cells in a dose-dependent manner compared with the untreated control culture (Figure 2a,d). The number of ingested *L. amazonensis* per macrophage was higher in the control group than that in cells incubated with 32 µM, but it was not different from the other PSN-1 concentrations (Figure 2b). The infection index, calculated as the average number of ingested amastigotes multiplied by the percentage of infected cells, was higher in the control than that in cells treated with any PSN-1 concentration (Figure 2c). The treatment for 2 h with 1, 8 or 32 μM of PSN-1 reduced the infection index of macrophages by 52%, 81% or 96%, respectively. The number of non-infected macrophages in relation to PSN-1 concentration followed a logarithmic function trend (Figure 2d).

**Figure 3:**
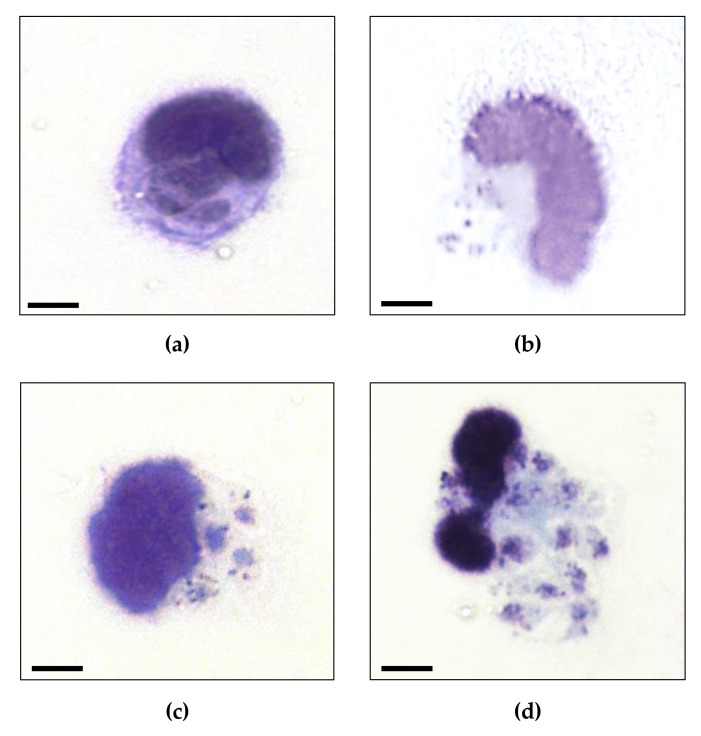
Photomicrography of amastigotes of *Leishmania amazonensis* inside infected-peritoneal macrophages non-treated (**a**) or treated with 1 (**b**), 8 (**c**) or 32 (**d**) µM phylloseptin-1 (PSN-1). Non-normal morphology of amastigotes in (**b**), (**c**) and (**d**); normal morphology of amastigotes in the non-treated cells (**a**). Cells were stained with Giemsa. Magnification = 1000 ×, Zeiss Axio Lab.A1 microscope. Scale bar = 4 µm.

Infected macrophages after 2 h of treatment with 8 or 32 μM of PSN-1 are shown in Figure 3. Light microscopy evaluation showed normal morphology of amastigote in (a), while in (b), (c) and (d) amastigotes did not show homogeneous aspect. This appearance suggests that amastigotes are in process of disintegration. Probably, these fragments are parasite debris, suggesting PSN-1 have a lytic effect on the amastigote forms of *L. amazonensis*.

**Figure 4:**
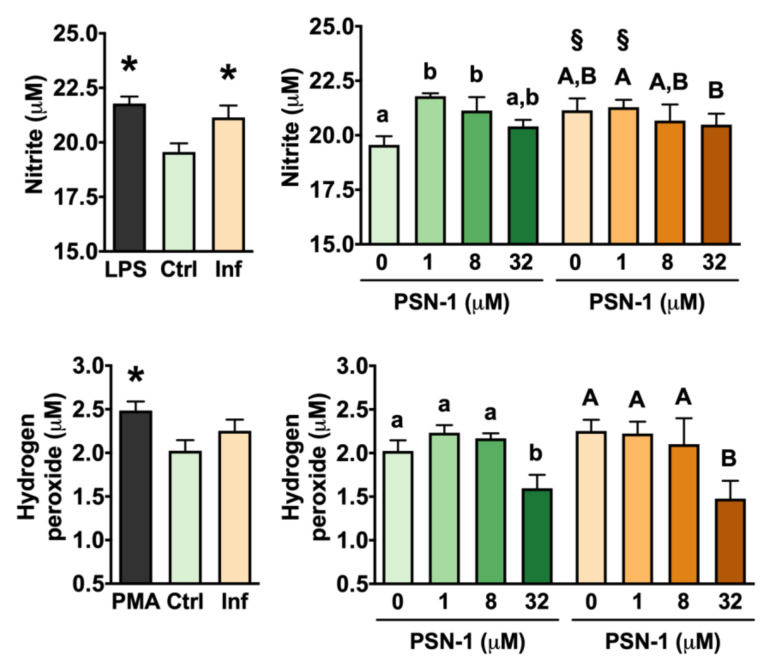
Nitric oxide production as estimated by nitrite measurement (top) and hydrogen peroxide levels (bottom) in peritoneal macrophages infected with axenic amastigotes of *Leishmania amazonensis* treated with different concentrations (0, 1, 8 or 32 μM) of phylloseptin-1 (PSN-1). Gray columns, positive control; green columns, uninfected macrophages; orange columns, infected macrophages. Data are presented as mean ± SEM (*n* = 7–8 repeated experiments). * Significantly different from the control group (unpaired *t-test*; *p* < 0.05). ^§^ Significantly different from the respective uninfected group (unpaired *t*-test; *p* < 0.05). Different lower case (uninfected) or upper case (infected) letters (i.e., groups that do not share any letter) indicate significant differences (one-way ANOVA followed by Tukey’s multiple comparisons test; *p* < 0.05).

Nitric oxide is a small, gaseous radical, produced enzymatically in macrophages from L-arginine and O_2_ by nitric oxide synthase. Nitrite is produced after reaction of NO with oxygen radicals and is assessed as an indirect measure of NO production [26,28,29,30,31]. Macrophages infected with *L. amazonensis* amastigotes without any treatment produced higher levels of NO than control non-infected cells (Figure 4) as indicated by higher nitrite levels. Similarly, stimulation with lipopolysaccharides elicited an increase in NO levels compared with control levels. In uninfected cells, PSN-1 slightly increased NO levels by 11% and 8% at 1 and 8 µM, respectively (Figure 4). In the case of hydrogen peroxide levels, there was no difference between infected and uninfected macrophages that were not treated with PSN-1 (Figure 4). Expectedly, exposure to PMA induced H_2_O_2_ production in macrophages (Figure 4). PSN-1 treatment had a significant effect on H_2_O_2_ levels only at the highest concentration (32 µM), when it reduced H_2_O_2_ concentration by 18% and 24% in uninfected and infected macrophages, respectively, compared with control levels (Figure 4).

**Figure 5:**
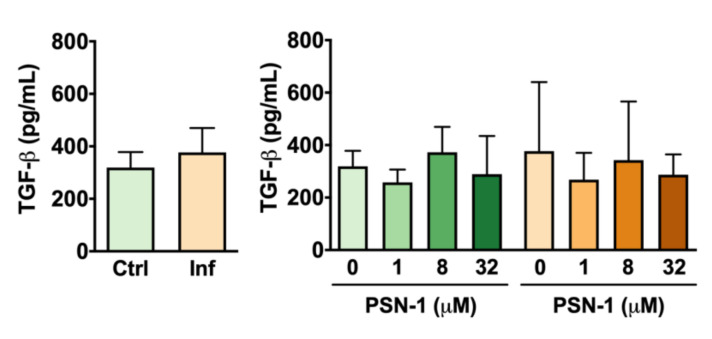
Transforming growth factor beta (TGF-β) levels (pg/mL) in the supernatant of peritoneal macrophages infected with axenic amastigotes of *Leishmania amazonensis* treated with different concentrations (0, 1, 8 or 32 μM) of phylloseptin-1 (PSN-1). Green columns, uninfected macrophages; orange columns, infected macrophages. Data are presented as mean ± SEM (*n* = 7–8 repeated experiments). No significant differences were detected.

Although infection caused an increase in the variability of data on TGF-β release by peritoneal macrophages, no differences were found between infected and uninfected macrophages or among macrophages treated with different concentrations of PSN-1 (Figure 5).

**Figure 6:**
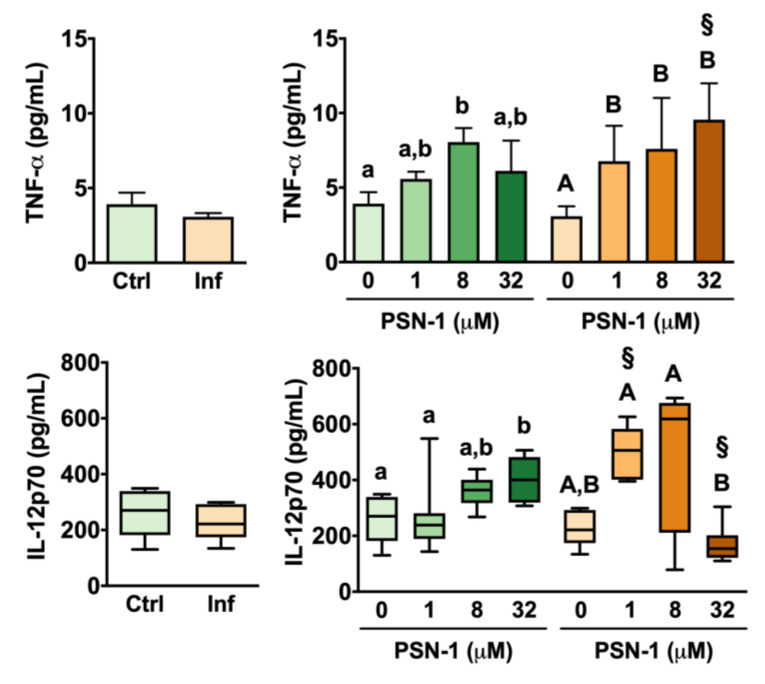
Tumor necrosis factor alpha (TNF-α; top) and interleukin-12 (IL-12; bottom) levels (pg/mL) in the supernatant of peritoneal macrophages infected with axenic amastigotes of *Leishmania amazonensis* treated with different concentrations (0, 1, 8 or 32 μM) of phylloseptin-1 (PSN-1). Green columns, uninfected macrophages; orange columns, infected macrophages. Data are presented as mean ± SEM (*n* = 6–8 repeated experiments) for TNF-α and as box plot for IL-12 (*n* = 7–8 repeated experiments). ^§^ Significantly different from the respective uninfected group (unpaired *t*-test; *p* < 0.05). Different lower case (uninfected) or upper case (infected) letters (i.e., groups that do not share any letter) indicate significant differences (one-way ANOVA followed by Tukey’s multiple comparisons test; *p* < 0.05).

Amastigote infection alone did not cause significant changes in levels of TNF-α released by macrophages (Figure 6). Among uninfected cells, PSN-1 treatment at 8 µM increased TNF-α levels significantly (by 105%) compared with the untreated group (Figure 6). At high (32 µM) and low (1 µM) doses, however, PSN-1 treatment had no significant effect on TNF-α release compared with the group of cells treated with vehicle only (Figure 6).

In the case of infected macrophages, TNF-α levels increased by 120% in the group treated with 1 µM compared with the untreated infected group (Figure 6). TNF-α release was also significantly higher in infected macrophages treated with 8 and 32 µM than in infected macrophages treated with vehicle only (Figure 6). When infected and uninfected groups were compared, it was found that, at 32 µM, infected macrophages had 56% more TNF-α than uninfected cells treated with the same concentration (Figure 6).

Infection by *L. amazonensis* amastigotes did not cause significant changes in IL-12 levels released by peritoneal macrophages without treatment with PSN-1 (Figure 6). Among uninfected groups, PSN-1 treatment at 32 µM caused a 56% increase in IL-12 levels compared with macrophages treated with vehicle only (Figure 6). This same concentration did not cause any significant change in IL-12 release by infected macrophages. In fact, when macrophages were treated with 32 µM PSN-1, IL-12 release was 132% higher in uninfected than in infected cells (Figure 6). In contrast, IL-12 levels were 87% higher in infected macrophages treated with 1 µM PSN-1 than in uninfected macrophages treated with 1 µM PSN-1 (Figure 6).

## 4. Discussion

Leishmaniasis still represents an important public health concern worldwide. People affected by this disease are usually the poorest, and the occurrence of leishmaniasis has been associated with environmental changes, such as deforestation and urbanization. Available treatments to combat *Leishmania* parasites are scarce and widely known to be associated with severe side effects. As an attempt to develop novel pharmacological treatments, we had previously shown that the peptide PSN-1 has leishmanicidal activity against *L. amazonensis* promastigotes [17]. In the present study, we expanded our knowledge on the leishmanicidal activity of PSN-1 against *L. amazonensis*, this time using their intracellular amastigote form. We found that PSN-1 had a strong dose-dependent leishmanicidal effect on amastigote-infected macrophages.

During the biological cycle of *Leishmania*, soon after inoculation of promastigotes by phlebotomine into the host, the macrophage infection occurs. Then, these flagellated forms begin their transformation, within the parasitophorous vacuoles, to their non-flagellated forms amastigotes. Such change allows the avoidance of macrophage microbicide mechanisms. Therefore, it is key to target parasites in their amastigote form. Our results showed that the microbicidal effect of PSN-1 occurred in the three evaluated concentrations through the evaluation of the infection index of macrophages, when compared with macrophages without treatment. These concentrations caused a significant reduction in the percentage of infected cells, without causing apparent damage to macrophages, as observed by morphological aspect of these cells and by previous evaluation of functional responses after treatment with PSN-1 [10,17]. This agrees with previous studies that have shown the absence of toxicity of PSN-1, in vivo, by its intravenous administration at 480 µg/mL to Swiss mice [10] or in vitro treatment of peritoneal cells with concentrations higher than 200 µg/mL [17].

The membrane of mammalian cells is mainly composed of neutral phospholipids with a localized positive charge due to the presence of ionized groups [32], which reduce their interaction with cationic peptides. Although reduced, the interaction of cationic peptides with cell membranes may occur, but especially with increasing concentration of the peptide, causing structural disruption and increased permeability of these cell membranes [33,34,35]. Toxic effects of antimicrobial peptides related to their interaction to the cell membrane have been shown for melittin from bee [36], caribdoxin present in the venom of scorpions [37] and the temporin-L, from frogs of the genus Rana [38]. Although PSN-1 is a cationic peptide, we found no signs of significant damage to macrophages suggested by morphological aspect and previous observation [10,17].

The morphological effects of PSN-1 on amastigotes suggest that the interaction of PSN-1 with the parasite occurred within the parasitophorous vacuoles. Possibly, the peptide initially interacted, by an unknown mechanism, with the cell membranes of macrophages and subsequently with the cell membrane of the parasite, as occurs for other antimicrobial peptides [39]. The antimicrobial activity of cationic peptides such as PSN-1 has been associated to the presence of phospholipids heavily loaded with positive charges, which allow them to interact with cell membranes of microorganisms, which commonly exhibit negative charges. Such interaction leads to changes in sodium and potassium flux and consequent increase in cell volume and disruption of the parasite cell [9,40,41,42,43,44]. Although we have not directly evaluated the mechanisms of action of PSN-1, we may speculate that the cellular debris observed within the macrophages may be a result from the formation of pores on the cell membrane as barrel-stave model [45]. According to this model, the pores are formed by the accumulation of monomeric peptides at a specific point of the membrane culminating with the drilling of the lipid bilayer [45].

The decrease in the infection rate in the cells treated with different concentrations of PSN-1 may also be explained by the interference of the peptide with the metabolic pathways of the parasite. For example, three analogs of magainin (MG-H1,-H2 and MG-F5W magainin-2) showed that these peptides decrease the production of ATP in a dose-dependent pattern in cultures of *Leishmania donovani*, causing the death of parasites [43]. Thus, it is possible that PSN-1 impaired oxidative phosphorylation and ATP production, leading to the shutdown of the metabolic processes and consequently the death of amastigotes; however, this explanation still requires experimental evidence.

To understand the molecular changes associated with the leishmanicidal effect of PSN-1 against *L. amazonensis* amastigotes, levels of key cytokines (TGF-β, TNF-α and IL-12) and the production of reactive species (H_2_O_2_ and NO) were assessed. These parameters were chosen based on the fact that the type of disease caused by *Leishmania* species depends on the genetics and immune response of the host. Parasites can trigger different host immune responses. The outcome of *Leishmania* infection depends on the development of polarized Th1 associated with resistance or Th2 responses with susceptibility. M1 macrophage and Th1 lymphocyte arm of immune response are associated with hydrogen peroxide and nitric oxide production. These two reactive species appear to be the main molecules involved in immune defense of the host. Inhibition of nitric oxide and oxygen radicals may be escape mechanisms of the parasite for their survival [46,47,48,49,50,51].

The increase in TNF-α indicates that PSN-1 also modulated the immune response. TNF-α enhances the defense response of macrophages against *Leishmania* [52]. Thus, it can be suggested that the increase in TNF-α release caused by PSN-1 might have participated in the destruction of the amastigotes inside macrophages observed in this work. It was also observed that PSN-1 upregulated IL-12p70 production in non-infected and up- and down-modulated IL-12 p70 production in infected macrophages, depending on the concentration of PSN-1. This cytokine stimulates the Th1 immune response, which is the main type of immune response against *Leishmania* [53], suggesting that in vivo treatment with PSN-1 might improve an adaptive immune defense against the parasite. However, infected macrophages treated with 32 µM PSN-1 decreased their IL-12p70 production. The cause of this effect is unclear, but some hypotheses may be considered. Cytokines are important modulators of an array of inflammatory and homeostatic processes. IL-10 is a potent negative regulator of IL-12, and activation of macrophages may stimulate regulatory mechanisms including the production of IL-10 [54,55]. Thus, the decrease in IL-12p70 production might be part of regulatory mechanisms of macrophages after infection that depended on PSN-1 concentration. Other possibility could be the stimulation via Toll-like receptor signaling by amastigotes of *Leishmania* combined with the concomitant treatment with high concentration of PSN-1, resulting in inhibition of IL-12p70 production instead of stimulation [56,57].

Noteworthy, at 32 µM (64 µg/mL), PSN-1 decreased H_2_O_2_ levels in both infected and uninfected macrophages. This indicates the potential antioxidant activity of this peptide in vivo. Nitric oxide and hydrogen peroxide are key players in the *Leishmania* killing process [30]; however, these molecules may also induce oxidative damage, so that these oxygen and nitrogen radicals may also contribute to the immunopathogenesis of leishmaniasis [52]. Treatment of amastigote-infected macrophages with PSN-1 decreased the production of such reactive molecules. Since PSN-1 has a potent leishmanicidal effect, it may be suggested that these immunomodulatory effects of the drug may favor the host instead of the parasite, by down-modulating the pathogenesis of the disease while the drug kill the parasite. Therefore, the mechanism of action of PSN-1 on *Leishmania* parasites and on innate and adaptive immune response of the host still warrants further research.

## 5. Conclusions

The effect of PSN-1 on amastigote forms of *L. amazonensis* further supports the potential use of the peptide for treatment of leishmaniasis. Its microbicidal effect occurred in a dose-dependent pattern, in which the maximum concentration (32 µM; 64 µg/mL) was able to reduce by 96% the infection index of macrophages. Although promising, further studies, in vitro and in animal models, are needed to clarify the mechanisms of action of PSN-1 and its interaction with the immune system.
